# Supplementary material for: Dynalign II: common secondary structure prediction for RNA homologs with domain insertions
Source: Nucleic Acids Res. 2014 Nov 21;42(22):13939–48. doi: 10.1093/nar/gku1172 (PMC4267632; doi:10.1093/nar/gku1172)
Supplement: SUPPLEMENTARY DATA [file supp_gku1172_nar-02021-z-2014-File012.zip › manual/RNA_class/html/annotated.html]

RNAstructure Classes: Class List

- Main Page
- Classes
- Files

- Class List
- Class Hierarchy
- Class Members

# Class List

Here are the classes, structs, unions and interfaces with brief descriptions:

|  |  |
| --- | --- |
| Dynalign\_object | Dynalign\_object Class |
| HybridRNA | HybridRNA Class |
| Multifind\_object | Multifind\_object Class |
| Multilign\_object | Multilign\_object Class |
| Oligowalk\_object | Oligowalk\_object Class |
| RNA | RNA Class |
| Thermodynamics | Thermodynamics Class |
| TwoRNA | TwoRNA Class |

---

Generated on Tue Jul 30 10:52:29 2013 for RNAstructure Classes by 
 1.5.7.1
